# Coverage, Trends, and Inequalities of Maternal, Newborn, and Child Health Indicators among the Poor and Non-Poor in the Most Populous Cities from 38 Sub-Saharan African Countries

**DOI:** 10.1007/s11524-023-00806-y

**Published:** 2023-12-18

**Authors:** Cauane Blumenberg, Janaina Calu Costa, Luiza I. Ricardo, Choolwe Jacobs, Leonardo Z. Ferreira, Luis Paulo Vidaletti, Fernando C Wehrmeister, Aluisio J. D. Barros, Cheikh Faye, Ties Boerma

**Affiliations:** 1 International Center for Equity in Health, Federal University of Pelotas, Pelotas, Brazil; 2 Post-Graduate Program in Epidemiology, Federal University of Pelotas, 1160 Marechal Deodoro St., 3rd floor, CEP 96020-220, Center, Pelotas, RS Brazil; 3 Causale consultoria, Pelotas, Brazil; 4 Harvard University, Boston, MA USA; 5 School of Public Health, University of Zambia, Lusaka, Zambia; 6 African Population and Health Research Center, Dakar, Senegal; 7 Institute for Global Public Health, University of Manitoba, Winnipeg, Canada

**Keywords:** Urbanization, Poverty, Inequalities, Sub-Saharan Africa

## Abstract

Rapid urbanization is likely to be associated with suboptimal access to essential health services. This is especially true in cities from sub-Saharan Africa (SSA), where urbanization is outpacing improvements in infrastructure. We assessed the current situation in regard to several markers of maternal, newborn, and child health, including indicators of coverage of health interventions (demand for family planning satisfied with modern methods, at least four antenatal care visits (ANC4+), institutional birth, and three doses of DPT vaccine[diphtheria, pertussis and tetanus]) and health status (stunting in children under 5 years, neonatal and under-5 mortality rates) among the poor and non-poor in the most populous cities from 38 SSA countries. We analyzed 136 population-based surveys (year range 2000–2019), contrasting the poorest 40% of households (referred to as poor) with the richest 60% (non-poor). Coverage in the most recent survey was higher for the city non-poor compared to the poor for all interventions in virtually all cities, with the largest median gap observed for ANC4+ (13.5 percentage points higher for the non-poor). Stunting, neonatal, and under-5 mortality rates were higher among the poor (7.6 percentage points, 21.2 and 10.3 deaths per 1000 live births, respectively). The gaps in coverage between the two groups were reducing, except for ANC4, with similar median average annual rate of change in both groups. Similar rates of change were also observed for stunting and the mortality indicators. Continuation of these positive trends is needed to eliminate inequalities in essential health services and child survival in SSA cities.

**Supplementary Information:**

The online version contains supplementary material available at 10.1007/s11524-023-00806-y.

## Introduction

The urban population is growing rapidly worldwide. In 2008, for the first time, more people were living in urban areas than in rural areas [1]. This phenomenon is mainly concentrated in towns and cities from low- and middle-income countries (LMICs), which will account for an estimated 95% of the urban population growth by 2030 [1]. When urbanization outpaces the improvements in infrastructure, as is the current situation in most LMICs, it typically has deleterious impacts on economic growth and inequalities [2]. This is especially concerning in sub-Saharan Africa (SSA), where most migrants to large cities end up residing in informal settlements and slums [3, 4].

Informal settlements and slums are characterized by overcrowding and suboptimal availability of basic services, such as adequate water, sanitation, and electricity [5]. In addition to the substandard built environment, which already puts urban dwellers from informal settlements and slums at disadvantage, these settings are also characterized by unsafe natural environments, inferior socio-economic resources, and unresponsive governance [6]. These aspects contribute to the surge of multidimensional intra-urban inequalities that have impacts on health and well-being. In terms of health, inequitable access to quality health services contributes to urban dwellers having disproportionally worse health outcomes [7]. The COVID-19 pandemic was the most recent example, as over 90% of all confirmed cases worldwide came from urban areas, which underscored the fact that large cities are the epicenters of most infections [8].

Understanding urban health systems, along with their inequalities, is complex. They can be described as open adaptive systems with multiple relationships that include health services, urban dwellers, and other different systems (e.g., ecological, political, social, and economic) [9]. Abejirinde and colleagues^1^ reviewed the literature to identify frameworks used to better understand and analyze urban health systems in the sub-Saharan African context. Of the five approaches identified, one centers on the comparison of urban health indicators with national, rural, and other urban areas from different countries or regions for highlighting and quantifying health inequalities [6]. However, this approach has rarely been used, as only a few studies perform comparisons between subgroups of the urban population, which could unveil inequalities at the intra-urban level.

Poverty has been described as an important aspect to be analyzed at the intra-urban level in regard to health care services and outcomes as it can contribute to the lack of access and utilization of quality reproductive, maternal, newborn, and child health (RMNCH) services, with the urban poor at increased risk for communicable and non-communicable diseases [10, 11].

To contribute to the knowledge and understanding around urban health systems, our objective is to examine the trends and the most recent situation of RMNCH indicators comparing poor and non-poor groups in the most populous cities of 38 SSA countries.

## Methods

### Data

Our analyses relied on two sources of nationally representative data, namely, Demographic and Health Surveys (DHS) and Multiple Indicator Cluster Surveys (MICS). Both DHS and MICS are cross-sectional surveys that use two-stage cluster sampling to select women of reproductive age (15 to 49 years) and children under 5 years. Due to their similar sampling methods and standardized questionnaires, DHS and MICS are considered highly comparable, being representative at national, regional, and residence (rural/urban) levels [12]. Although both surveys cover entire countries, our analyses were restricted to the results for the most populous city in each country. These were identified from city population sizes obtained from the Population Division of the United Nations Department of Economic and Social Affairs [1].

For trend analyses, we included countries with two or more national surveys available since 2000 and which had the most recent survey conducted from 2010 onwards. Countries with a single survey conducted after 2010 were included for the analyses of the most recent situation. Furthermore, only the surveys that had the city of interest as a sample domain were eligible for the analyses. In some surveys, the most populous city was part of a domain that covered a wider geographical area. In these cases, the surveys were included if the target city’s population represented 70% or more of the whole domain population. This was the case for five countries: Benin, Burkina Faso, Madagascar, Namibia, and Sierra Leone. Detailed information for these settings is provided in Supplementary Table 1. These domains were treated as if they were the cities of interest.

### Health Indicators

Four health intervention coverage indicators were studied, covering four steps of the RMNCH continuum of care: demand for family planning satisfied with modern methods (mDFPS), at least four antenatal care visits (ANC4+), institutional delivery, and child immunization with three doses of DPT (diphtheria, pertussis, and tetanus) vaccine (DPT3). mDFPS was defined as the proportion of women of reproductive age (15–49 years of age) who were sexually active (married, in a union, or sexually active); in need of contraception (fecund women who did not want or were unsure about becoming pregnant in the next 2 years); and who were using (or whose partner was using) modern contraceptive methods (sterilization, subdermal implants, intrauterine devices, oral contraceptives, condoms, emergency contraceptive pills, injectables, vaginal rings, and patches) [13]. ANC4+ was the proportion of women of reproductive age who had a birth in the last 2 (MICS surveys) or 3 years (DHS surveys) with at least four ANC visits with any provider. Institutional delivery was defined as the proportion of all live births in the last 3 or 5 years (MICS and DHS, respectively) that occurred in a health facility. Finally, DPT3 was the proportion of live children aged 12–23/18–29/15–26 months (according to the city’s vaccination calendar) who received three doses of the DPT vaccine.

Also, health status indicators were explored: the prevalence of stunting and childhood mortality measured through neonatal mortality rates (NMR) and under-5 mortality rates (U5MR). Stunting was defined as children from 0 to 59 months of age who were below −2 standard deviations from the median height for age. NMR and U5MR were calculated by dividing the number of deaths at age 0–30 days and 0–59 months, respectively, by the number of surviving children at the beginning of the age range in the 10 years preceding the survey.

### Dimension of Inequality

Inequalities in RMNCH indicators were assessed by comparing each city’s poor and non-poor groups. Both groups were defined according to households’ socioeconomic position based on the survey-specific asset index [14, 15]. This index is calculated through a principal component analysis (PCA) using the ownership of household appliances, characteristics of the building, and other relevant economic variables as predictors [16].

Using the continuous national wealth index provided in each survey, households from each city of interest were divided into poor, the 40% of households with the lowest scores, and non-poor, the remaining 60% (proportions weighted by the number of household members). The cut-off to define poor and non-poor was chosen based on previous studies [17, 18] and to ensure sufficient sample size in the poor and non-poor groups for all cities analyzed [19].

### Statistical Analyses

The most recent situation regarding the selected outcomes was described for each city’s poor and non-poor groups based on estimates for the latest survey available, totaling 38 cities and surveys (Table 1). We visually presented inequalities between the city poor and non-poor using equiplots (https://equidade.org/equiplot), which enable the visualization of both the estimates in each group and the gaps between them (absolute inequality). Absolute inequality indicators were calculated by the difference between poor and non-poor groups.

**Table 1:** List of cities and surveys included in the analyses sorted by population size

City	Country	City’s population size (in millions)	Number of surveys	Survey year range
Lagos	Nigeria	14.37	3	2007, –11, –16
Kinshasa	DR Congo	13.17	4	2007, –10, –13, –17
Luanda	Angola	7.77	1	2015
Dar es Salaam	Tanzania	6.70	3	2004, –10, –15
Khartoum	Sudan	5.53	2	2010, –14
Ville d’Abidjan	Côte d’Ivoire	4.92	3	2006, –11, –16
Addis Ababa	Ethiopia	4.40	5	2000, –05, –11, –16, –19
Nairobi	Kenya	4.39	3	2003, –08, –14
Douala	Cameroon	3.66	5	2004, –06, –11, –14, –18
Kampala	Uganda	2.99	3	2006, –11, –16
Dakar	Senegal	2.98	9	2005, –10, –12, –14–19
Ouagadougou ^a^	Burkina Faso	2.53	3	2003, –06, –10
Lusaka	Zambia	2.52	4	2001, –07, –13, –18
Greater Accra	Ghana	2.51	6	2003, –06, –08, –11, –14, –17
Bamako	Mali	2.45	6	2001, –06, –09, –12, –15, –18
Brazzaville	Congo	2.23	3	2005, –11, –14
Conakry	Guinea	1.84	4	2005, –12, –16, –18
Lomé Commune	Togo	1.75	4	2006, –10, –13, –17
Harare	Zimbabwe	1.52	6	2005, –09, –10, –14–15, –19
N’Djaména	Chad	1.32	4	2004, –10, –14, –19
Greater Antananarivo ^a^	Madagascar	1.28	3	2003, –08, –18
Niamey	Niger	1.21	3	2006, –12, –21
Nouakchott	Mauritania	1.21	4	2007, –11, –15, –19
Freetown ^a^	Sierra Leone	1.14	6	2005, –08, –10, –13, –17, –19
Maputo Cidade	Mozambique	1.10	4	2003, –08, –11, –15
Kigali Ville	Rwanda	1.06	4	2000, –10, –14, –19
Lilongwe City	Malawi	1.03	1	2013
Bujumbura	Burundi	0.90	2	2010, –16
Bangui	CAR	0.85	3	2006, –10, –18
Libreville, Port-Gentil	Gabon	0.81	2	2000, –12
Cotonou ^a^	Benin	0.69	4	2006, –11, –14, –17
Ngazidja ^a^	Comoros	0.40	1	2012
Windhoek ^a^	Namibia	0.40	3	2000, –06, –13
SAB - Setor Autônomo de Bissau ^b^	Guinea-Bissau	0.39	3	2006, –14, –18
Kanifing	Gambia	0.38	5	2005, –10, –13, –18–19
Maseru	Lesotho	0.20	3	2004, –09, –14
Distrito de Água Grande	São Tomé and Príncipe	0.07	1	2019
Manzini	Eswatini	0.03	3	2006, –10, –14

^a^Surveys covered a larger region in which the most populous city in the country represented 70% or more of the whole domain’s population

^b^Autonomous region that was considered as the most populous city in the country

Trends were evaluated by estimating the average annual rate of change (AARC) for the selected outcomes. AARC is largely used to assess changes in prevalence over the years, taking into consideration the period between the baseline to the most recent survey in each city [20, 21]. Second degree fractional polynomials were fitted to assess departures from linearity for each indicator and group (city poor and non-poor). Based on deviance reduction, exponential models did not show significant improvements compared to linear models. Thus, the latter were used in the analyses. To estimate AARC, the indicator estimates were log transformed and regressed against the survey years using ordinary least-squares (OLS) models. Then, AARC was calculated by the formula *AARC* = (1 − *e*^*β*^) ×  − (1.) Positive AARC values indicate the average yearly percentage by which the indicator is increasing, while negative AARC values indicate the rate of decrease. Thirty-four cities (132 surveys) with two or more surveys available could have had trends estimated (Table 1).

Statistical differences between the estimates for the poor and non-poor groups were assessed by overlapping estimates and 95% confidence intervals. All analyses were conducted using Stata® software version 17.0 (StataCorp LLC, College Station, TX, USA) and have considered the sampling design including clustering and sample weights.

## Results

The analyses included almost 47,000 children and 27,000 women residing in the most populous cities from 38 sub-Saharan African countries (equivalent to 79% of all countries in the region). The median population size of cities included in the analyses was 1.42 million, ranging from 0.03 million in Manzini (Eswatini) to 14.37 million in Lagos (Nigeria) (Table 1).

### Most Recent Situation

**Fig. 1:**
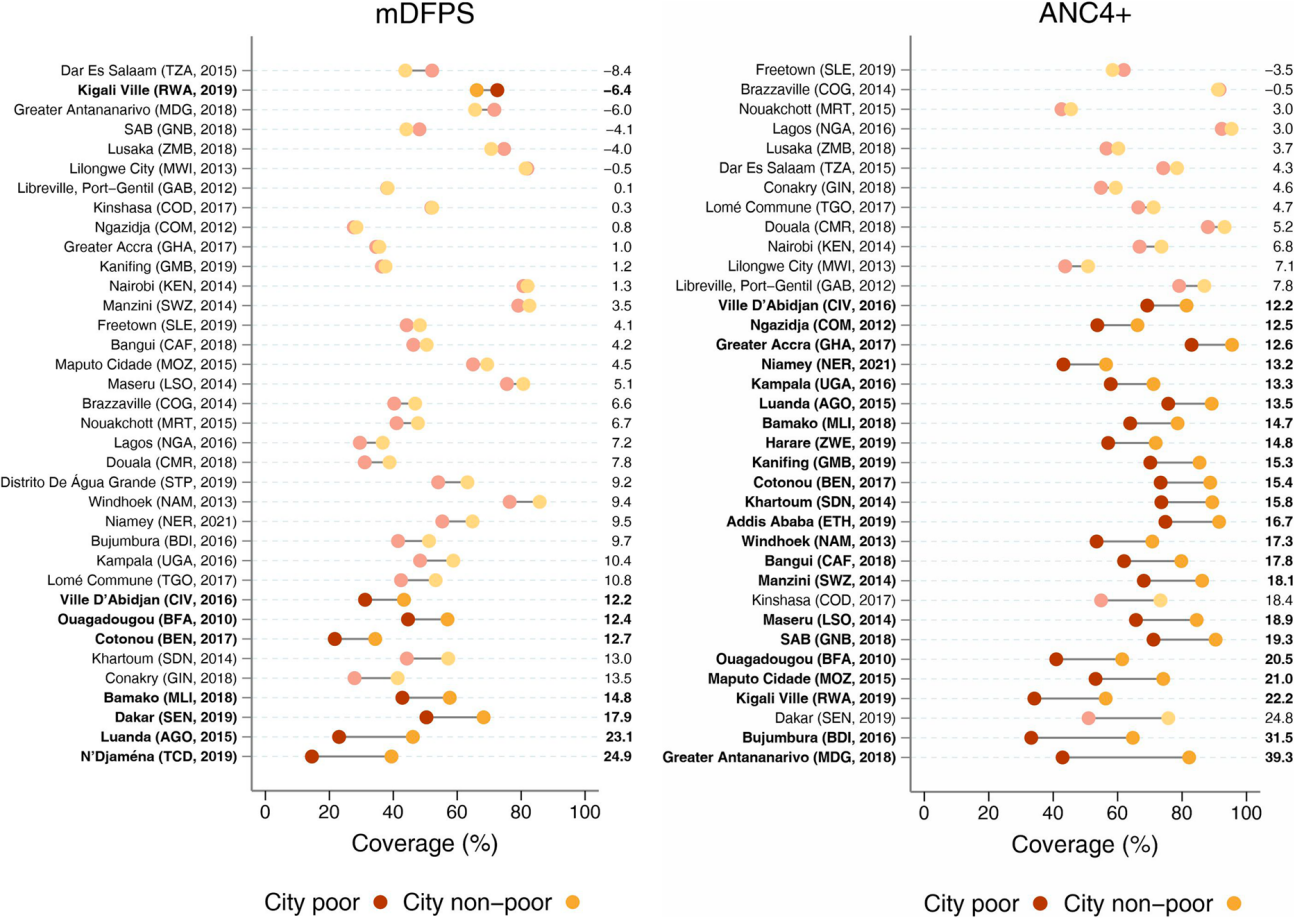
Coverage of demand for family planning satisfied with modern methods (mDFPS) (left) and at least four antenatal care visits (ANC4+) (right) in the latest survey from each city according to poor and non-poor groups. Note: Numbers on the right represent the absolute gap comparing the coverage of the non-poor to the poor, with solid colors representing statistically significant differences

Median mDFPS coverage was 48.3% for all cities analyzed, and the median gap between the cities’ non-poor and poor was 6.6 percentage points (p.p.). N’Djaména (Chad) showed the widest gap, with coverage of 24.9 p.p. higher among the non-poor (39.4% coverage). Kigali Ville (Rwanda) was the only city with a reversed pattern, in which the mDFPS coverage was 6.4 p.p. higher among the poor (72.5 coverage) (Fig. 1).

Coverage of ANC4+ had a higher median estimate (71.1%) and a median non-poor vs. poor gap of 13.5 p.p. This gap ranged from −3.5 p.p. in Freetown (Sierra Leone) to 39.3 p.p. in Greater Antananarivo (Madagascar) (Fig. 1). More than three-fifths (61%) of all cities showed statistically significant differences, all in favor of the non-poor.

**Fig. 2.**
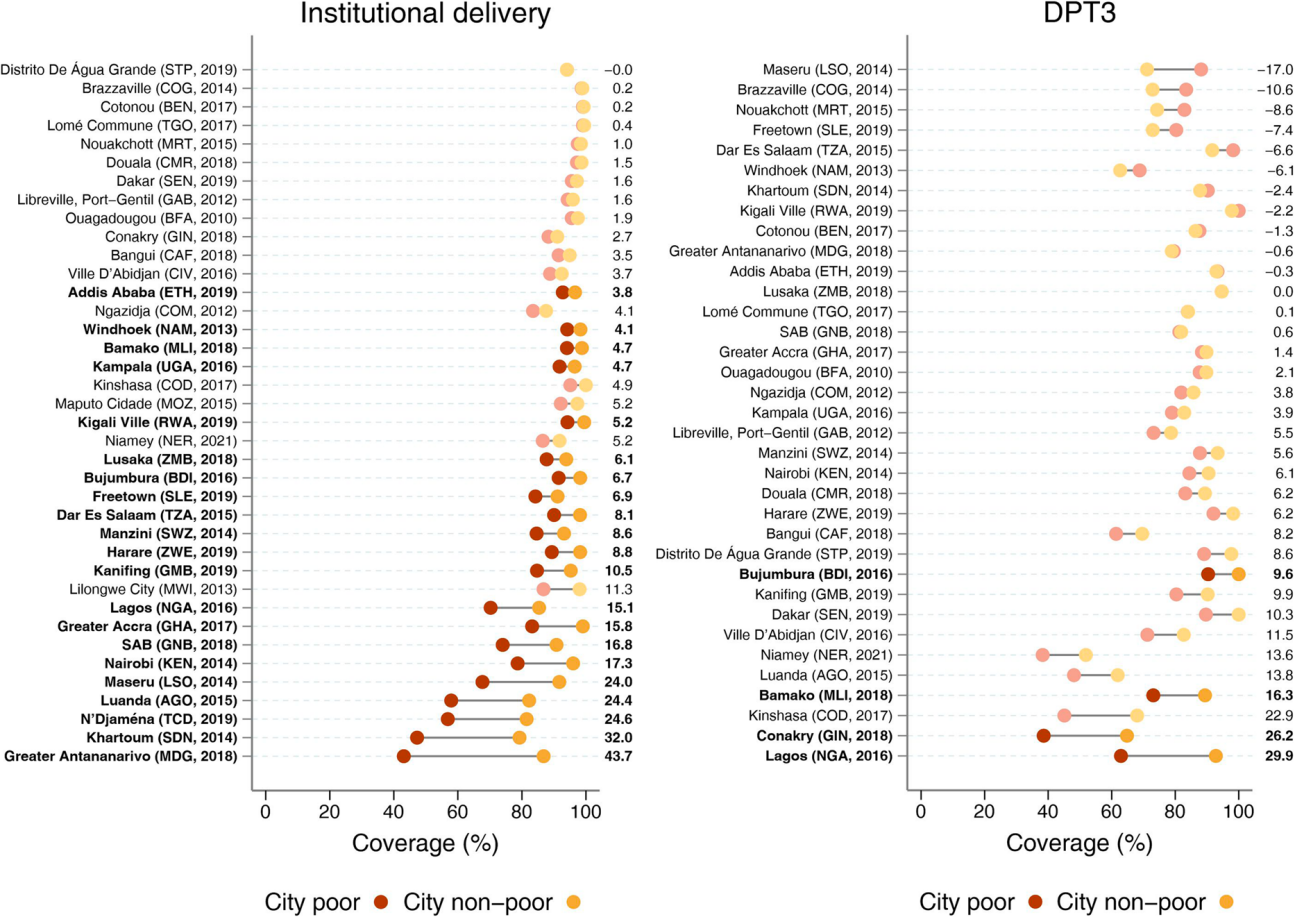
Coverage of institutional delivery (left) and three doses of DPT3 vaccine (right) in the latest survey from each city according to city poor and non-poor groups. Note: Numbers on the right represent the absolute gap comparing the coverage of the non-poor to the poor, with solid colors representing statistically significant differences

Median coverage of institutional delivery across all cities was 93.5%, and the level was higher than 80% among the poor in 30 cities (79% of all cities). The median non-poor vs. poor gap was 5.2 p.p., with inequalities in most cities being lower than 10 p.p. Greater Antananarivo (Madagascar) showed the greatest inequalities (43.7 p.p. gap in favor of the non-poor, which had 87% coverage) (Fig. 2).

Regarding DPT3, more diverse results were found. The median coverage of DPT3 was 84.0%, with a median non-poor vs. poor gap of 3.9 p.p. Differences between the groups ranged from −17 p.p. in Maseru (Lesotho) to 29.9 p.p. in Lagos (Nigeria). This was the intervention with the largest number of cities with estimates favoring the poor groups, a pattern observed in 10 out of the 32 settings analyzed. In 25 out of the 32 cities, DPT3 vaccination coverage was 80% or higher among the poor (Fig. 2).

**Fig. 3:**
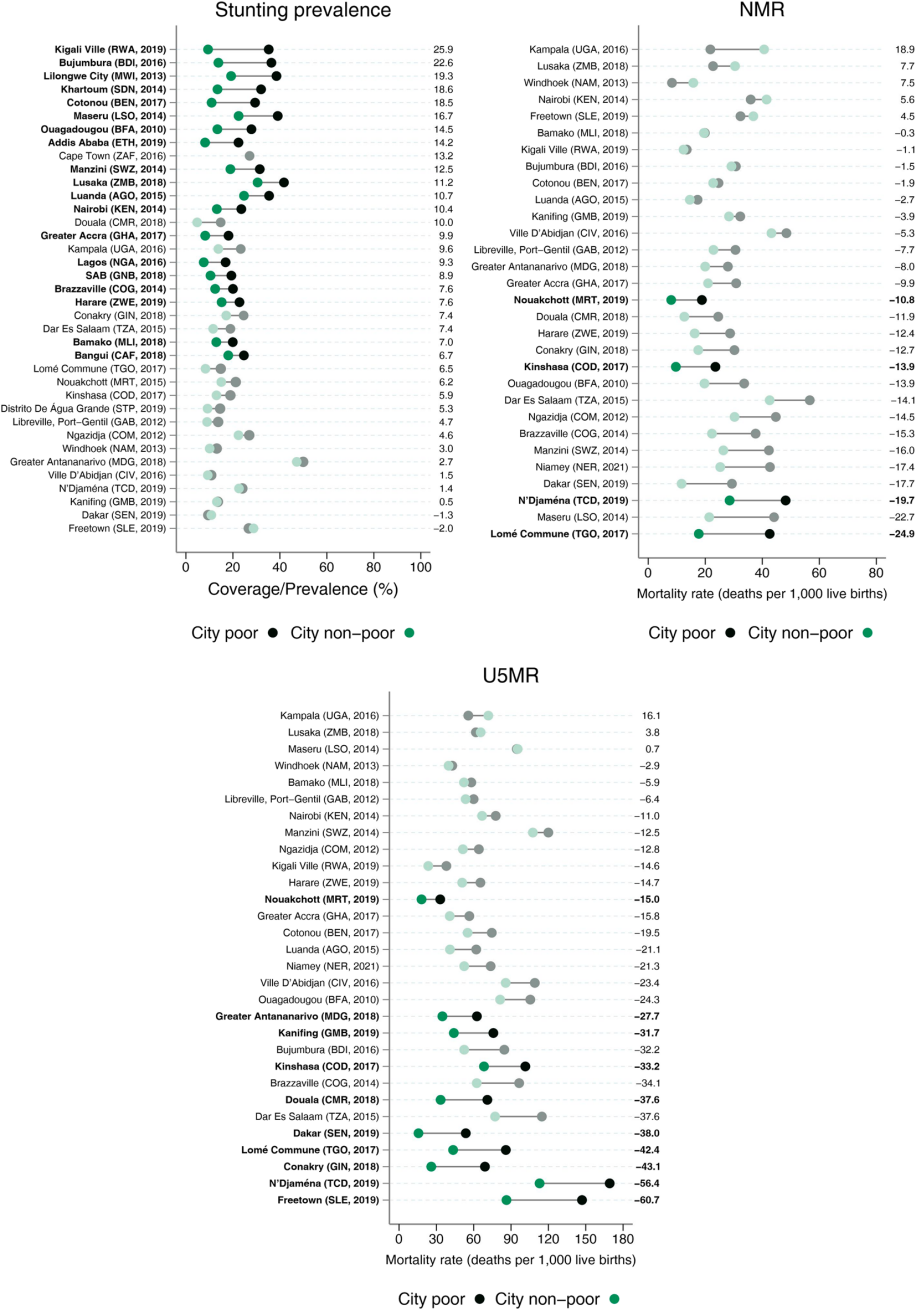
Stunting prevalence (left), NMR (right), and U5MR (bottom) in the latest survey from each city according to poor and non-poor groups. Note: Numbers on the right represent the absolute gap comparing the coverage of the non-poor to the poor, with solid colors representing statistically significant differences

Median stunting prevalence was 23% among the city poor and 13% among the non-poor. Stunting prevalence was higher among the poor in all but two cities (Dakar (Senegal) and Freetown (Sierra Leone)). We observed a median gap of −7.6 p.p. between non-poor and poor, with only two cities presenting difference higher than 20 p.p.: Kigali Ville (Rwanda) and Bujumbura (Burundi) (Fig. 3).

The median U5MR gap comparing the non-poor to the poor across all 38 cities was −21.2 deaths/1000 live births. It varied considerably among cities, ranging from −60.7 deaths/1000 live births in Freetown (Sierra Leone) to 16.1 deaths/1000 live births in Kampala (Uganda). Only three cities had U5MR lower than 25/1000 live births among the city non-poor (Dakar (Senegal), Kigali Ville (Rwanda), and Nouakchott (Mauritania)), and only three cities had lower U5MR among the poor compared to the non-poor (Kampala (Uganda), Lusaka (Zambia), and Maseru (Lesotho)) (Fig. 3).

Similar patterns were found regarding neonatal mortality, although with smaller differences. The median NMR gap across all 38 cities was −10.3 deaths/1000 live births, ranging from −24.9 p.p. in Lomé Commune (Togo) to 18.9 p.p. in Kampala (Uganda). Three cities (Dakar (Senegal), Kinshasa (DR Congo), and Nouakchott (Mauritania)) had NMR lower than 12/1000 live births among the non-poor, and Windhoek (Namibia) had 8.3 deaths per 1000 live births among the city poor (Fig. 3).

### Trend Analyses

Considering all 34 cities with two or more surveys, the median AARC for mDFPS, institutional delivery, and DPT3 was twice as large in the city poor compared to the non-poor (Table 2). Institutional delivery was the coverage indicator with most cities reducing the gap between the poor and non-poor (41.2% of the cities). AARC for the city poor and non-poor groups were similar for ANC4+ (AARC_poor_ = 0.5%; AARC_non-poor_ = 0.2%), with a similar proportion of cities reducing and widening the gaps (11.8%).

**Table 2:** Median average annual rate of change (AARC) and respective interquartile range (IQR) for coverage and impact indicators. Proportion of cities reducing and widening inequality gaps is also presented

	Median AARC (IQR)		Cities reducing the gap (%)^a^	Cities widening the gap (%)^b^
	Poor	Non-poor		
mDFPS	2.4 (0.1; 5.3)	1.2 (−1.4; 4.9)	29.4	8.8
ANC4+	0.5 (−0.8; 1.4)	0.2 (−0.5; 1.1)	11.8	11.8
Institutional delivery	1.1 (0.3; 2.5)	0.5 (0.1; 1.2)	41.2	8.8
DPT3	1.1 (−0.2; 1.8)	0.4 (−1.2; 1.4)	23.5	5.9
Stunting	−2.2 (−4.0; −1.5)	−3.3 (−5.9; −1.0)	5.9	11.8
NMR	−1.3 (−3.1; 1.0)	−1.5 (−4.8; 0.7)	16.0	32.0
U5MR	−3.3 (−5.1; −1.9)	−3.2 (−4.9; −1.5)	28.0	32.0

*AARC*, average annual rate of change; *ANC4+*, at least four antenatal care visits; *DPT3*, three doses of diphtheria, pertussis, and tetanus vaccine; *IQR*, interquartile range; *mDFPS*, demand for family planning satisfied with modern methods; *NMR*, neonatal mortality rate; *U5MR*, under-5 mortality rate

^a^Reducing gap between poor and non-poor (AARC poor > non-poor for coverage indicators, AARC poor < non-poor for impact indicators)

^b^Widening gap between poor and non-poor (AARC poor < non-poor for coverage indicators, AARC poor > non-poor for impact indicators)

**Fig. 4:**
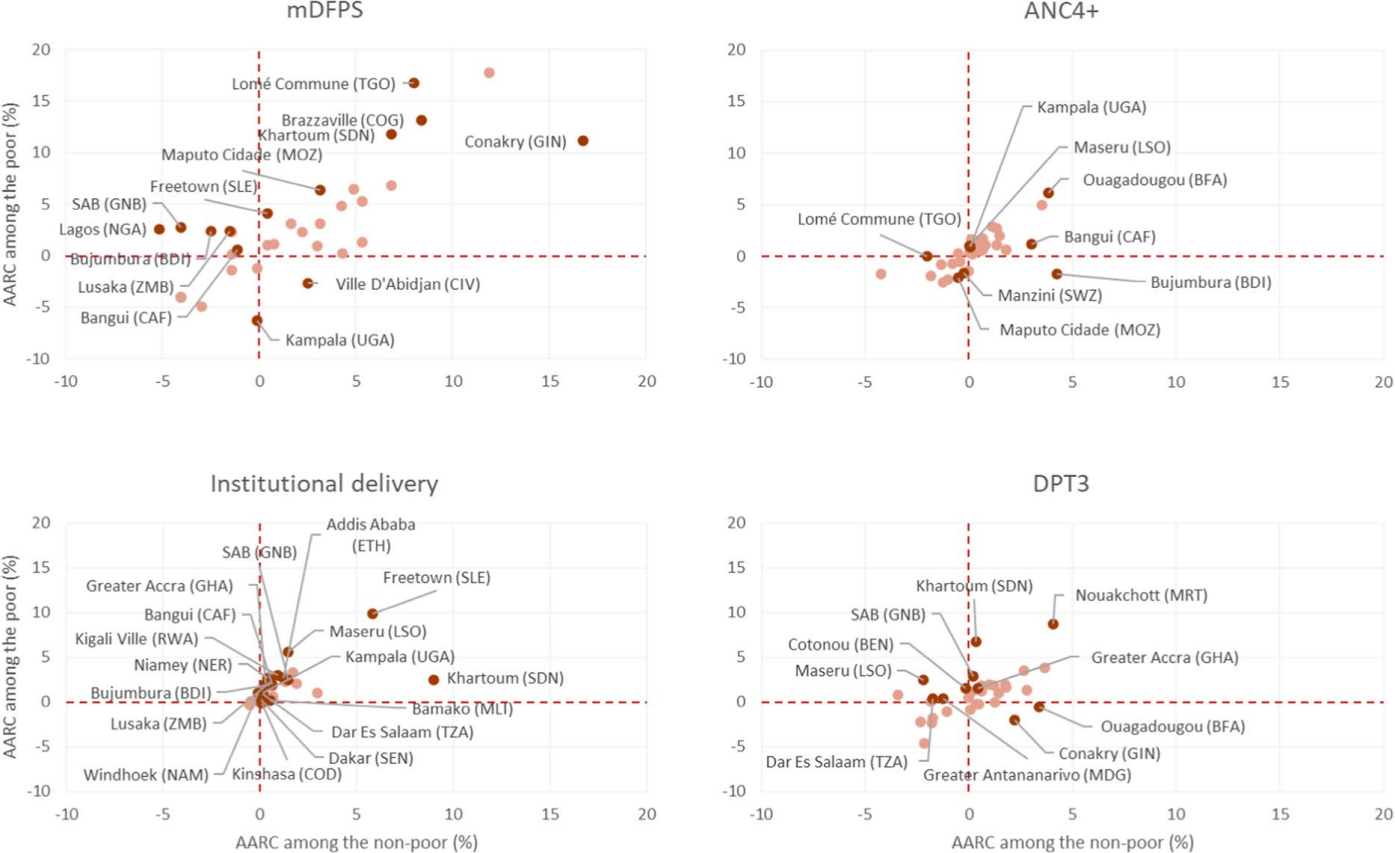
Average annual rate of change (AARC) in coverage indicators for the poor and non-poor groups from each city. Note: Dark red color represents statistically significant differences comparing the poor to non-poor. ANC4+, at least four antenatal care visits; DPT3, three doses of diphtheria, pertussis, and tetanus vaccine; mDFPS, demand for family planning satisfied with modern methods

Rates of change in mDFPS were statistically different between the poor and non-poor in 13 cities (38% of those included in the analyses) (solid colors in Fig. 4). Ten out of the 13 cities had higher AARC among the city poor compared to the non-poor. The largest increase in coverage among the poor was observed in Lomé Commune (Togo); it was twice as large among the non-poor (AARC = 16.7% vs. 8.0%, respectively). Eight cities presented contrasting AARC estimates (e.g., increasing trend among the city poor and decreasing among the non-poor, or the opposite). Ville d’Abidjan (Côte d’Ivoire) was the only city where mDFPS coverage among the poor reduced, by 2.7%, while also increasing among the non-poor (by 2.5%) (Fig. 4 and Supplementary Table 3).

AARC in coverage of ANC4+ was higher among the city poor compared to the non-poor only in four cities (Kampala (Uganda), Lomé Commune (Togo), Maseru (Lesotho), and Ouagadougou (Burkina Faso)) (Fig. 4). Ouagadougou (Burkina Faso) was the city with the highest AARC among the poor, 6.1% (1.6 times higher than the non-poor). Two other cities presented steeper decreasing trends in coverage among the poor compared to the non-poor (Manzini (Eswatini) and Maputo Cidade (Mozambique)) (Fig. 4 and Supplementary Table 3).

Regarding institutional delivery, a decreasing coverage trend among the non-poor was observed only from Lagos (Nigeria) (AARC_non-poor_ = −0.5%). This was the coverage indicator with the largest number of cities with statistical differences between the AARC from the poor and non-poor; such differences were observed in half (*N*=17) of the cities analyzed (Fig. 4). The highest AARC among the city poor was observed in Freetown (Sierra Leone) (AARC_poor_ = 9.9%), while among the non-poor, the highest was in Khartoum (Sudan) (AARC_non-poor_ = 9.0%) (Supplementary Table 1). Ten cities (29% of the cities analyzed) had different trends on the coverage of DPT3 between the city poor and non-poor (Fig. 4). Conakry (Guinea) was the only city with a decreasing AARC among the poor and increasing among the non-poor (AARC_poor_ = −2.0%; AARC_non-poor_ = 2.2%) (Supplementary Table 3).

**Fig. 5:**
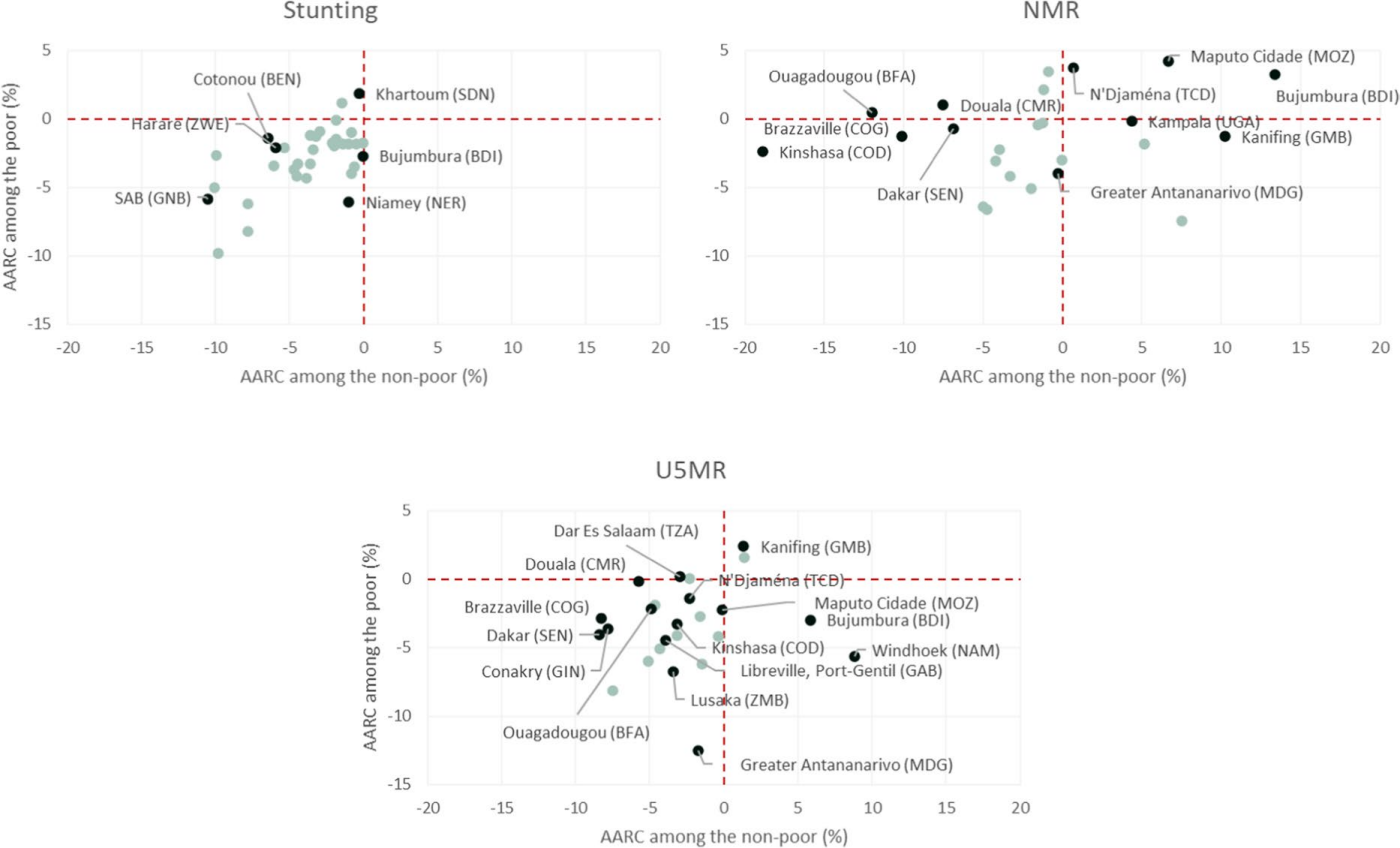
Average annual rate of change (AARC) in the prevalence of stunting, neonatal mortality, and under-5 mortality rates among the poor and non-poor groups from each city. Note: Dark green color represents statistically significant differences comparing the city poor to non-poor. NMR, neonatal mortality rate; U5MR, under-5 mortality rate.

Regarding impact indicators (stunting, NMR, and U5MR), the median AARC among the poor was similar compared to the non-poor, all with decreasing rates. However, the proportion of cities widening inequality gaps between the poor and non-poor was twice as large compared to those reducing the stunting and NMR gaps (Table 2). Stunting AARC comparing the city poor to non-poor was statistically different in six cities, with steeper reductions among the poor only in Bujumbura (Burundi) and Niamey (Niger) (Fig. 5). Among the city poor, increasing trends were observed in Khartoum (Sudan) (AARC_poor_ = 1.9%) and Ouagadougou (AARC_poor_ = 1.1%) (Supplementary Table 3).

For U5MR, the median AARC for the poor and non-poor also showed a similar proportion of cities reducing and widening the inequality gaps (Table 2). The largest decrease was observed in Greater Antananarivo (Madagascar) (12.5% and 7.4 times higher compared to the city non-poor). The non-poor from Windhoek (Namibia) had an AARC compatible with an 8.9% increase in U5MR per year, contrasting with an average reduction of 5.6% among the poor from the same city (Fig. 5 and Supplementary Table 3).

Detailed AARC estimates with accompanying 95% confidence intervals of each coverage and impact indicator for each city and according to city poor and non-poor groups are provided in Supplementary Table 3 and Supplementary Table 3.

## Discussion

We analyzed the most populous city from 38 sub-Saharan African countries comparing the coverage of interventions and health status between the city poor and non-poor groups. Our results showed that, in general, coverage of interventions was higher among the city non-poor than among the poor—with some cities showing pronounced gaps between the two groups. Prevalence of stunting and mortality rates were higher among the city poor than among the non-poor. Trend analyses showed steeper increases in coverage of interventions among the city poor compared to the non-poor (except for ANC4+) and similar trends for stunting and mortality estimates.

Considering the lower coverage of interventions among the city poor compared to the non-poor, and the faster coverage increases among the former, a reduction in the gap between poor and non-poor is ongoing and seems likely to continue. This pattern is in line with the inverse equity hypothesis, which postulates that the better-off benefit first from life-saving interventions, until they reach adequate coverage [22]. Subsequently, the pace among the poorer populations starts to increase, reducing the gap between the groups and, eventually, also reaching higher coverage levels. In most cities, coverage of institutional delivery and DPT3 among the poor had already reached 80% coverage, and further gap reductions are likely to be as they get close to near-universality.

The high levels of institutional delivery among the city poor, which were similar to the non-poor estimates in most cities, contrast with the higher levels of NMR among the poor compared to the non-poor in the same city. In general, coverage of institutional deliveries is inversely associated both with neonatal and maternal mortality. It is estimated that universal institutional delivery could avert around 60% of all neonatal deaths and would be even more effective among the poor compared to the non-poor [23–26]. Similarly, over 30% of all maternal deaths could be avoided with universal facility-based births [27]. Thus, our results show somewhat of a paradox, with high levels of institutional delivery appearing to have limited effect on reducing neonatal mortality. This is in line with the recent findings regarding maternal mortality, which have stagnated globally despite increases in institutional delivery [28].

It is important to note that giving birth in a health facility might not be sufficient to reduce neonatal and mortality deaths if the facilities lack quality and content of care. The type and preparedness of the health facility where the birth took place is an important aspect. As Gage and colleagues showed,^2^ the share of hospital deliveries in sub-Saharan Africa was associated with lower neonatal mortality, while the share of deliveries in any facility was not [24]. Also of importance are the quantity and quality of health workers in health facilities, which can further contribute to the reduction of maternal and neonatal deaths [29]. Further analyses that study deliveries by type of facility and health workers who assisted during labor, which signal the content of care provided, may throw further light on this issue.

The coverage of ANC4+ was near-universal in 18 groups from 15 cities. However, out of the 18 groups, only four were poor. Taking into consideration that the increasing trend on ANC4+ coverage among the city poor was only slightly faster than among the non-poor, inequalities on antenatal care are likely to persist in most SSA cities. When considering both urban and rural areas, the literature shows varying results. A study of 35 SSA countries found coverage of ANC4+ ranging from 32 to 92% in both urban and rural areas [30]. Also, socioeconomic inequalities were observed. Another study of 31 SSA countries showed that women from the richest households had 2.41 increased odds of adequately accessing antenatal care when compared to the poorest group. Also, this likelihood was 54% higher among urban women compared to rural [31].

Regarding mDFPS, only five cities had coverage higher than 80% among the poor or non-poor. Nairobi (Kenya) and Lilongwe City (Malawi) were the only cities where mDFPS coverage was higher than 80% both among the poor and non-poor. In 1967, in an effort to slow down its rapid population growth, Kenya was the first SSA country to develop a national family planning program. Between 1978 and 1998, the country was able to reduce the total fertility rate from 8.1 to 4.7 children per woman [32, 33]. In 2010, the right to access to good reproductive health care was included in the Kenyan constitution, and in 2011, the government started covering all costs related to contraception [32]. Malawi’s government also has made substantial commitments to improve family planning nationwide. It added a specific family planning line to the country’s budget, which has contributed to investment increasing by 10 times in a 7-year period (reaching nearly US$200 million in 2020, which is expected to double by 2023) [34]. The impacts include improvements in the supply chain and in the offer of a mix of contraceptive methods, including self-injection and youth-friendly family planning strategies. Through such efforts, Malawi was able to increase modern contraceptive prevalence from 7 to 58% between the 1990s and 2016 [34, 35].

The under-5 mortality target of the Sustainable Development Goals (SDGs) is 25 per 1000 live births by 2030 [36]. Three cities among the 38 analyzed have already achieved it, as indicated in the most recent survey estimates: Dakar (Senegal) (16/1000 live births), Nouakchott (Mauritania) (18/1000 live births), and Kigali Ville (Rwanda) (24/1000 live births). Studies in Rwanda and Senegal attributed the progress to policies that focused on primary health care and increasing vaccination coverage [37]. Across all 38 cities, none of the poor groups had yet achieved the under-5 mortality target. For all to meet these targets, a median annual reduction of 47 and 29 deaths per 1000 live births among the city poor and non-poor, respectively, would be needed.

Dakar (Senegal), Nouakchott (Mauritania), Windhoek (Namibia), and Kinshasa (DR Congo) have already achieved the SDG target for neonatal mortality rates below 12 deaths per 1000 live births [36]. However, Windhoek was the only city where the target has been met for the poor as well as the non-poor. Across all 38 cities, the poor and non-poor from cities that did not reach the target would need, respectively, a median reduction of 23 and 18 deaths per 1000 live births. The situation comparing the city poor and non-poor is less unequal regarding NMR than U5MR. This is partly due to the determinants of mortality. NMR and U5MR are both influenced by socioeconomic factors and the availability and quality of health care; however, neonatal mortality is mainly influenced by pregnancy complications and birth-related aspects [38, 39].

Some limitations regarding our paper are important to note. Mortality rates in our study were based on the full reproductive history of the sampled women and calculated according to DHS recommendations, reflecting estimates that consider events over the 10 years preceding the surveys [40, 41]. Thus, our estimates might not accurately reflect the current situation in the cities analyzed. However, this approach was necessary to guarantee that mortality estimates were reliable due to the reduced sample size when analyzing cities instead of the whole sample and to allow stratification by wealth groups. A gap persists in terms of understanding the impacts of the COVID-19 pandemic on current mortality estimates. To fill this gap, mortality-focused surveys enabling disaggregation by subgroups of the population are needed. Ideally, they should cover moments before and after the pandemic to quantify its impacts on neonatal and under-5 mortality. Regarding the analytical approach, the multi-city analysis was beneficial to give an overview of many of the largest SSA cities; however, the wide range of survey years available make comparisons challenging. The endline years of some surveys were the baseline ones for others. Thus, when comparing the most recent situation among cities, it is important to take the survey year into consideration. This drawback was reduced in our trend analyses, as we took into account baseline estimates by calculating AARC estimates [20]. Finally, the city poor definition we used—comparing the 40% poorest to the 60% wealthiest—is not the only definition available in the literature [5]. The main reason behind choosing this cut-off was related to guaranteeing sufficient sample size to run analyses for all cities [19]. As our focus was to give an overview of SSA cities, this was the most suitable definition to be used, but context-specific definitions should be employed when focusing on a single city.

Our results suggest that there is suboptimal access to and coverage of health interventions among the poorer from the most populous cities in SSA, findings that in turn reflect on higher prevalence and rates of stunting and mortality among children. However, our findings showed that in general across the cities, the gap between the city poor and non-poor is closing for most health interventions, although not for the impact indicators analyzed. In order to see improvements in all aspects of urban health, it is important to adopt approaches that recognize African cities as inequitable places, but also places that are central to efforts to achieve the SDGs agenda by 2030 [42]. The availability of intersectoral, longitudinal, and disaggregated data to monitor and better understand the urban health system failures that contribute to health inequalities is vital for more rapid progress toward the SDGs [42, 43]. Our results contribute to the understanding of intra-urban health inequalities according to one dimension (wealth) and considering a multi-city approach. However, other inequality dimensions and city-specific approaches could be studied to provide in-depth information to policy makers for designing context-specific multisectoral strategies to improve access to good-quality RMNCH services for those most in need.

## Supplementary Information

Below is the link to the electronic supplementary material.

ESM 1 (DOCX 54.7 KB)

## Footnotes

1 Abejirinde I-OO, Gwacham-Anisiobi U, Affun-Adegbulu C, Vanhamel J, Van Belle S, Marchal B. A perspective on urban health systems and research for equitable healthcare in Africa. *BMJ Global Health*. 2022;7:e010333.

2 Gage AD, Fink G, Ataguba JE, Kruk ME. Hospital delivery and neonatal mortality in 37 countries in sub-Saharan Africa and South Asia: An ecological study. Myers JE, editor. *PLOS Med*. 2021;18:e1003843.
