# Supplementary material for: Coverage, Trends, and Inequalities of Maternal, Newborn, and Child Health Indicators among the Poor and Non-Poor in the Most Populous Cities from 38 Sub-Saharan African Countries
Source: J Urban Health. 2023 Dec 18;101(Suppl 1):31–44. doi: 10.1007/s11524-023-00806-y (PMC11602901; doi:10.1007/s11524-023-00806-y)
Supplement: Supplementary file 1 — (DOCX 54.7 KB) [file 11524_2023_806_MOESM1_ESM.docx]

# Coverage, trends, and inequalities of maternal, newborn, and child health indicators among the poor and non-poor in the most populous cities from 38 sub-Saharan African countries

# Supplementary tables

Supplementary table 1. List of surveys in which the region was sampling domain, but it was considered as the most populous city from the country as at least 70% of the region's population is represented by the city's population.

| **Country** | **Region** | **Region's population (in millions)** | **Most populous city** | **City's population (in millions)** | **% of region population represented by the city's population** | **Surveys with this pattern** |
| --- | --- | --- | --- | --- | --- | --- |
| Benin | Littoral | 0.68 | Cotonou | 0.68 | 100% | DHS 2006, -11, -17; MICS 2014 |
| Burkina Faso | Centre | 1.70 | Ouagadougou | 1.48 | 87% | DHS 2010; MICS 2006 |
| Madagascar | Analamanga | 3.60 | Greater Antananarivo | 3.00 | 83% | DHS 2008; MICS 2018 |
| Namibia | Khomas | 0.48 | Windhoek | 0.43 | 90% | DHS 2000, -06, -13 |
| Sierra Leone | West | 1.49 | Freetown | 1.06 | 71% | DHS 2008, -13, -19; MICS 2005, -10, -17 |

Supplementary table 2. Average annual rate of change (AARC) and respective confidence intervals for coverage of interventions in each city according to urban poor and non-poor groups. Abbreviations: 95% CI: 95% confidence interval; ANC4+: at least four antenatal care visits; DPT3: three doses of diphtheria, pertussis, and tetanus vaccine; mDFPS: demand for family planning satisfied with modern methods.

| **City** | **mDFPS** | | | | **ANC4+** | | | | **Institutional delivery** | | | | **DPT3** | | | |
| --- | --- | --- | --- | --- | --- | --- | --- | --- | --- | --- | --- | --- | --- | --- | --- | --- |
|  | **City poor** | | **City non-poor** | | **City poor** | | **City non-poor** | | **City poor** | | **City non-poor** | | **City poor** | | **City non-poor** | |
|  | **AARC** | **95% CI** | **AARC** | **95% CI** | **AARC** | **95% CI** | **AARC** | **95% CI** | **AARC** | **95% CI** | **AARC** | **95% CI** | **AARC** | **95% CI** | **AARC** | **95% CI** |
| Addis Ababa (ETH, 2000-19) | 0.1 | -0.2; 0.5 | -1.4 | -3.0; 0.2 | 1.5 | 0.2; 2.8 | 0.5 | 0.1; 0.9 | 2.7 | 1.9; 3.4 | 1.2 | 0.4; 2.0 | 1.2 | 0.2; 2.1 | 0.6 | -0.2; 1.5 |
| Bamako (MLI, 2001-18) | 4.9 | -1.3; 11.0 | 4.3 | -0.1; 8.6 | 0.5 | -1.0; 2.1 | 0.6 | -0.3; 1.4 | 0.2 | 0.0; 0.4 | 0.5 | 0.3; 0.8 | -0.2 | -0.7; 0.3 | 0.5 | -0.6; 1.6 |
| Bangui (CAF, 2006-18) | 0.6 | 0.2; 1.0 | -1.1 | -2.7; 0.4 | 1.2 | -0.5; 2.8 | 3.0 | 1.9; 4.2 | 1.9 | 1.0; 2.9 | 0.6 | 0.1; 1.2 | 1.3 | -2.1; 4.6 | 0.6 | -0.8; 1.9 |
| Brazzaville (COG, 2005-14) | 13.2 | 12.8; 13.5 | 8.4 | 6.8; 10.0 | 1.6 | -1.6; 4.8 | 0.1 | -0.6; 0.8 | 0.3 | 0.0; 0.5 | 0.4 | -0.1; 0.8 | 0.0 | -3.8; 3.9 | -1.8 | -1.8; -1.8 |
| Bujumbura (BDI, 2010-16) | 2.4 | 2.0; 2.8 | -2.5 | -4.1; -0.9 | -1.7 | -3.3; -0.1 | 4.3 | 3.1; 5.4 | 1.9 | 0.7; 3.2 | 0.6 | 0.4; 0.7 | 0.0 | -1.6; 1.5 | 1.3 | 0.4; 2.1 |
| Conakry (GIN, 2005-18) | 11.1 | 9.1; 13.2 | 16.7 | 13.3; 20.2 | -0.7 | -4.5; 3.1 | -0.8 | -4.5; 2.9 | 2.0 | 1.8; 2.3 | 1.9 | 1.4; 2.4 | -2.0 | -3.1; -0.9 | 2.2 | 0.7; 3.7 |
| Cotonou (BEN, 2006-17) | 1.3 | 0.0; 2.7 | 5.3 | -0.3; 10.9 | -1.4 | -3.7; 0.8 | 0.0 | -2.6; 2.6 | 0.0 | -0.3; 0.2 | 0.0 | -0.3; 0.2 | 1.5 | 0.0; 3.1 | -0.2 | -1.7; 1.4 |
| Dakar (SEN, 2005-19) | 6.5 | 0.5; 12.5 | 4.9 | 3.5; 6.4 | 2.7 | -0.5; 5.9 | 1.4 | 0.4; 2.3 | 0.3 | 0.1; 0.5 | 0.0 | -0.1; 0.2 | 1.9 | 0.9; 2.9 | 1.8 | 1.0; 2.5 |
| Dar Es Salaam (TZA, 2004-15) | 6.9 | 6.5; 7.2 | 6.9 | 5.3; 8.4 | -2.5 | -11.6; 6.5 | -1.2 | -1.5; -1.0 | 0.9 | 0.2; 1.6 | 0.2 | -0.1; 0.4 | 0.3 | -1.2; 1.9 | -1.7 | -2.6; -0.9 |
| Douala (CMR, 2004-18) | -1.2 | -8.6; 6.3 | -0.1 | -9.3; 9.1 | 0.3 | 0.0; 0.6 | 0.1 | -0.5; 0.7 | 0.1 | -0.1; 0.3 | 0.0 | -0.1; 0.2 | 1.8 | -0.3; 4.0 | 0.6 | -0.1; 1.4 |
| Freetown (SLE, 2005-19) | 4.1 | 0.4; 7.8 | 0.4 | -0.3; 1.1 | -0.8 | -3.6; 2.0 | -1.3 | -5.0; 2.3 | 9.9 | 6.4; 13.3 | 5.8 | 4.0; 7.6 | 1.1 | -0.3; 2.5 | 0.1 | -1.9; 2.1 |
| Greater Accra (GHA, 2003-17) | -4.9 | -7.2; -2.6 | -3.0 | -12.8; 6.8 | 1.4 | 0.4; 2.4 | 0.5 | -0.1; 1.1 | 2.0 | 1.1; 2.9 | 0.3 | 0.2; 0.5 | 1.5 | 0.8; 2.3 | 0.4 | -0.1; 1.0 |
| Greater Antananarivo (MDG, 2003-18) | -4.0 | -4.4; -3.7 | -4.0 | -5.6; -2.4 | 2.0 | -4.1; 8.1 | 1.5 | 0.3; 2.6 | 1.0 | -1.5; 3.5 | 3.0 | 1.8; 4.2 | 0.3 | -0.9; 1.6 | -1.2 | -1.5; -1.0 |
| Harare (ZWE, 2005-19) | 3.1 | -1.6; 7.7 | 1.6 | 0.9; 2.3 | 0.2 | -1.5; 1.9 | 0.2 | -2.6; 3.0 | 0.2 | -0.8; 1.3 | 0.5 | -0.1; 1.0 | 3.5 | 1.7; 5.2 | 2.6 | 1.3; 4.0 |
| Kampala (UGA, 2006-16) | -6.2 | -6.6; -5.9 | -0.1 | -1.7; 1.5 | 0.9 | 0.9; 1.0 | 0.1 | -0.5; 0.6 | 1.0 | 0.3; 1.7 | 0.2 | 0.0; 0.3 | 1.6 | -1.2; 4.5 | 1.8 | 0.4; 3.2 |
| Kanifing (GMB, 2005-19) | 2.3 | -1.1; 5.7 | -1.4 | -5.7; 2.9 | 0.6 | -0.6; 1.8 | 0.7 | 0.1; 1.3 | 0.5 | -0.4; 1.4 | 0.7 | 0.6; 0.8 | -0.2 | -1.2; 0.8 | 0.3 | -0.5; 1.2 |
| Khartoum (SDN, 2010-14) | 11.8 | 11.4; 12.1 | 6.9 | 5.3; 8.4 | 1.0 | -0.6; 2.7 | 1.3 | 0.2; 2.5 | 2.5 | 1.2; 3.7 | 9.0 | 8.9; 9.1 | 6.7 | 5.2; 8.3 | 0.3 | -0.5; 1.2 |
| Kigali Ville (RWA, 2000-19) | 1.1 | 0.1; 2.2 | 0.7 | 0.1; 1.4 | 5.0 | 4.3; 5.7 | 3.5 | 1.8; 5.3 | 3.0 | 2.2; 3.8 | 0.9 | 0.7; 1.2 | 1.8 | 0.7; 2.9 | 1.2 | 0.2; 2.2 |
| Kinshasa (COD, 2007-17) | 17.7 | 12.4; 23.0 | 11.9 | 2.3; 21.5 | -0.5 | -3.0; 1.9 | -0.4 | -1.7; 0.8 | -0.2 | -0.4; 0.1 | 0.1 | 0.0; 0.3 | -4.6 | -10.1; 0.9 | -2.2 | -4.6; 0.3 |
| Lagos (NGA, 2007-16) | 2.6 | 2.2; 3.0 | -5.2 | -6.7; -3.6 | 0.3 | -0.8; 1.4 | 0.0 | -0.2; 0.3 | 0.1 | -0.9; 1.0 | -0.5 | -0.6; -0.3 | 1.3 | -4.9; 7.6 | 2.8 | 2.1; 3.5 |
| Libreville, Port-Gentil (GAB, 2000-12) | -1.4 | -1.8; -1.1 | -1.4 | -3.0; 0.2 | 1.1 | -0.5; 2.7 | 0.8 | -0.3; 1.9 | 0.4 | -0.9; 1.6 | 0.0 | -0.1; 0.1 | 3.8 | 2.3; 5.4 | 3.6 | 2.8; 4.5 |
| Lomé Commune (TGO, 2006-17) | 16.7 | 9.2; 24.3 | 8.0 | 7.8; 8.2 | 0.0 | -0.3; 0.3 | -2.0 | -3.0; -1.1 | 0.6 | -0.5; 1.6 | 0.3 | 0.2; 0.5 | 2.0 | -1.8; 5.8 | 1.0 | -0.5; 2.5 |
| Lusaka (ZMB, 2001-18) | 2.4 | 2.1; 2.8 | -1.5 | -3.1; 0.1 | -1.9 | -5.4; 1.6 | -1.8 | -4.4; 0.7 | 2.2 | 1.1; 3.3 | 0.6 | 0.1; 1.1 | 0.4 | 0.0; 0.8 | 0.0 | -0.5; 0.5 |
| Manzini (SWZ, 2006-14) | 1.1 | 0.7; 1.4 | 0.4 | -1.1; 2.0 | -1.7 | -1.7; -1.6 | -0.2 | -0.6; 0.2 | 2.9 | 0.0; 5.7 | 0.6 | -0.5; 1.8 | -0.9 | -2.8; 1.1 | 0.1 | -1.5; 1.7 |
| Maputo Cidade (MOZ, 2003-15) | 6.4 | 6.1; 6.8 | 3.1 | 1.6; 4.7 | -2.1 | -3.3; -0.8 | -0.5 | -0.8; -0.2 | 0.5 | 0.2; 0.9 | 0.2 | -0.3; 0.6 | -1.1 | -2.8; 0.7 | -1.1 | -1.2; -0.9 |
| Maseru (LSO, 2004-14) | -4.0 | -4.4; -3.7 | -4.0 | -5.6; -2.4 | 1.0 | 0.7; 1.3 | 0.0 | -0.3; 0.3 | 5.6 | 3.9; 7.2 | 1.5 | 0.5; 2.4 | 2.4 | 1.0; 3.9 | -2.2 | -4.2; -0.2 |
| N'Djaména (TCD, 2004-19) | 0.2 | -15.2; 15.7 | 4.3 | -3.9; 12.5 | 0.4 | -0.7; 1.4 | 0.3 | -3.5; 4.0 | 3.3 | 0.9; 5.6 | 1.7 | 1.3; 2.1 | 0.8 | -27.5; 29.1 | -3.4 | -10.7; 3.9 |
| Nairobi (KEN, 2003-14) | -4.0 | -4.4; -3.7 | -4.0 | -5.6; -2.4 | 0.3 | -0.9; 1.4 | -0.5 | -1.3; 0.2 | 1.8 | -2.3; 5.9 | 0.7 | 0.6; 0.7 | 1.0 | -0.1; 2.2 | 1.4 | 1.1; 1.8 |
| Niamey (NER, 2006-21) | 2.3 | 1.9; 2.6 | 2.2 | 0.6; 3.8 | 2.9 | -1.9; 7.7 | 1.1 | 1.0; 1.2 | 2.7 | 0.8; 4.6 | 0.4 | -0.5; 1.2 | -1.9 | -11.9; 8.2 | -1.7 | -7.9; 4.4 |
| Nouakchott (MRT, 2007-2019) | 1.0 | -5.7; 7.7 | 3.0 | 0.2; 5.7 | -1.7 | -15.4; 11.9 | -4.2 | -16.4; 8.0 | 2.2 | 0.9; 3.5 | 1.3 | 0.5; 2.1 | 8.7 | 7.2; 10.2 | 4.1 | 0.6; 7.6 |
| Ouagadougou (BFA, 2003-10) | 5.3 | -0.3; 10.9 | 5.3 | -0.3; 10.9 | 6.1 | 4.5; 7.7 | 3.9 | 2.7; 5.0 | 1.2 | -16.5; 18.8 | 0.1 | -2.6; 2.8 | -0.5 | -2.1; 1.0 | 3.4 | 2.5; 4.2 |
| SAB (GNB, 2006-18) | 2.8 | 2.4; 3.1 | -4.0 | -5.6; -2.4 | 0.6 | -1.0; 2.2 | 1.8 | 0.6; 2.9 | 2.5 | 1.6; 3.4 | 1.5 | 1.0; 1.9 | 2.9 | 0.3; 5.5 | 0.2 | -1.5; 2.0 |
| Ville D'Abidjan (CIV, 2006-16) | -2.7 | -3.0; -2.3 | 2.5 | 0.9; 4.1 | 1.7 | 0.1; 3.3 | 0.7 | -0.5; 1.8 | -0.3 | -1.3; 0.7 | -0.5 | -1.2; 0.1 | -2.4 | -4.2; -0.5 | -1.8 | -3.6; 0.0 |
| Windhoek (NAM, 2000-13) | 3.1 | 2.8; 3.5 | 3.1 | 1.6; 4.7 | -2.2 | -3.9; -0.5 | -1.0 | -2.0; -0.1 | 1.0 | 0.0; 2.0 | -0.1 | -0.3; 0.2 | -2.2 | -2.4; -2.0 | -2.3 | -7.6; 3.0 |
| **Median AARC and IQR** | **2.4** | **0.1; 5.3** | **1.2** | **-1.4; 4.9** | **0.5** | **-0.8; 1.4** | **0.2** | **-0.5; 1.1** | **1.1** | **0.3; 2.5** | **0.5** | **0.1; 1.2** | **1.1** | **-0.2; 1.8** | **0.4** | **-1.2; 1.4** |

Supplementary table 3. Average annual rate of change (AARC) and respective confidence intervals for impact indicators in each city according to urban poor and non-poor groups. Abbreviations: 95% CI: 95% confidence interval.

| **City** | **Stunting** | | | | **Neonatal mortality rate** | | | | **Under-five mortality rate** | | | |
| --- | --- | --- | --- | --- | --- | --- | --- | --- | --- | --- | --- | --- |
|  | **City poor** | | **City non-poor** | | **City poor** | | **City non-poor** | | **City poor** | | **City non-poor** | |
|  | **AARC** | **95% CI** | **AARC** | **95% CI** | **AARC** | **95% CI** | **AARC** | **95% CI** | **AARC** | **95% CI** | **AARC** | **95% CI** |
| Addis Ababa (ETH, 2000-19) | -3.7 | -5.2; -2.3 | -4.7 | -5.6; -3.8 |  |  |  |  |  |  |  |  |
| Bamako (MLI, 2001-18) | -1.5 | -3.5; 0.5 | -1.9 | -4.4; 0.7 | -6.4 | -6.9; -6.0 | -5.0 | -7.3; -2.7 | -6.0 | -8.0; -4.0 | -5.1 | -7.7; -2.6 |
| Bangui (CAF, 2006-18) | -3.3 | -5.1; -1.5 | -3.6 | -5.3; -1.9 |  |  |  |  |  |  |  |  |
| Brazzaville (COG, 2005-14) | -2.6 | -9.2; 3.9 | -10.0 | -19.4; -0.6 | -1.3 | -3.1; 0.5 | -10.1 | -10.9; -9.4 | -2.9 | -2.9; -2.8 | -8.2 | -8.3; -8.2 |
| Bujumbura (BDI, 2010-16) | -2.7 | -3.0; -2.5 | 0.0 | -1.0; 0.9 | 3.3 | 1.4; 5.1 | 13.4 | 12.6; 14.1 | -3.0 | -3.1; -2.9 | 5.9 | 5.9; 5.9 |
| Conakry (GIN, 2005-18) | -1.8 | -5.5; 2.0 | -2.1 | -8.2; 4.0 | -3.1 | -3.2; -2.9 | -4.2 | -8.7; 0.2 | -3.7 | -5.2; -2.1 | -7.8 | -10.6; -5.1 |
| Cotonou (BEN, 2006-17) | -1.4 | -6.4; 3.5 | -6.4 | -7.4; -5.5 | -4.2 | -9.5; 1.2 | -3.3 | -12.2; 5.6 | -2.7 | -5.6; 0.2 | -1.6 | -8.0; 4.8 |
| Dakar (SEN, 2005-19) | -2.0 | -8.1; 4.1 | -2.0 | -6.3; 2.4 | -0.7 | -4.7; 3.3 | -6.8 | -12.8; -0.9 | -4.1 | -7.4; -0.8 | -8.4 | -12.4; -4.4 |
| Dar Es Salaam (TZA, 2004-15) | -3.3 | -4.3; -2.3 | -4.5 | -8.9; 0.0 | 5.8 | 5.0; 6.7 | -1.6 | -5.6; 2.4 | 0.2 | -0.6; 1.0 | -3.0 | -4.7; -1.2 |
| Douala (CMR, 2004-18) | -6.2 | -9.5; -2.9 | -7.8 | -10.5; -5.2 | 1.0 | -7.5; 9.5 | -7.5 | -12.1; -2.9 | -0.2 | -4.2; 3.8 | -5.7 | -8.9; -2.5 |
| Freetown (SLE, 2005-19) | -4.0 | -7.1; -0.9 | -0.8 | -4.6; 2.9 | -5.1 | -9.3; -0.9 | -2.0 | -9.0; 5.1 | -1.9 | -3.1; -0.7 | -4.6 | -8.5; -0.8 |
| Greater Accra (GHA, 2003-17) | -2.3 | -4.5; 0.0 | -3.4 | -8.0; 1.2 | -0.4 | -4.5; 3.7 | -1.5 | -8.0; 5.0 | -4.2 | -5.0; -3.3 | -3.2 | -12.4; 6.1 |
| Greater Antananarivo (MDG, 2003-18) | -1.8 | -4.1; 0.6 | 0.0 | -0.7; 0.7 | -4.0 | -5.8; -2.2 | -0.3 | -1.0; 0.5 | -12.5 | -12.6; -12.5 | -1.7 | -1.8; -1.7 |
| Harare (ZWE, 2005-19) | -2.1 | -5.0; 0.8 | -5.9 | -9.5; -2.3 | -0.3 | -6.6; 6.0 | -1.3 | -14.7; 12.1 | 0.0 | -5.2; 5.2 | -2.3 | -8.1; 3.5 |
| Kampala (UGA, 2006-16) | -1.3 | -9.5; 6.9 | -3.2 | -4.2; -2.2 | -0.2 | -0.7; 0.3 | 4.4 | 3.2; 5.5 | -6.2 | -7.9; -4.6 | -1.5 | -7.1; 4.1 |
| Kanifing (GMB, 2005-19) | -1.9 | -8.0; 4.3 | -0.5 | -4.7; 3.7 | -1.3 | -3.1; 0.5 | 10.2 | 9.5; 10.9 | 2.4 | 2.3; 2.4 | 1.3 | 1.3; 1.4 |
| Khartoum (SDN, 2010-14) | 1.9 | 1.6; 2.1 | -0.3 | -1.3; 0.6 |  |  |  |  |  |  |  |  |
| Kigali Ville (RWA, 2000-19) | -0.9 | -2.2; 0.4 | -2.9 | -6.2; 0.3 | -6.7 | -10.1; -3.2 | -4.8 | -8.3; -1.2 | -8.2 | -12.0; -4.3 | -7.5 | -8.6; -6.5 |
| Kinshasa (COD, 2007-17) | -4.2 | -7.2; -1.2 | -4.5 | -9.4; 0.4 | -2.4 | -4.2; -0.6 | -18.9 | -19.6; -18.2 | -3.3 | -3.4; -3.2 | -3.1 | -3.2; -3.1 |
| Lagos (NGA, 2007-16) | -5.0 | -19.3; 9.3 | -10.1 | -25.0; 4.8 |  |  |  |  |  |  |  |  |
| Libreville, Port-Gentil (GAB, 2000-12) | -4.3 | -4.6; -4.1 | -3.9 | -4.9; -2.9 | -2.2 | -4.1; -0.4 | -3.9 | -4.7; -3.2 | -4.5 | -4.6; -4.4 | -3.9 | -3.9; -3.9 |
| Lomé Commune (TGO, 2006-17) | -2.1 | -5.7; 1.5 | -5.3 | -6.2; -4.5 |  |  |  |  |  |  |  |  |
| Lusaka (ZMB, 2001-18) | -1.0 | -1.9; -0.1 | -0.8 | -1.1; -0.5 | -3.0 | -10.5; 4.5 | 0.0 | -0.8; 0.8 | -6.8 | -9.8; -3.7 | -3.4 | -5.7; -1.0 |
| Manzini (SWZ, 2006-14) | -1.2 | -4.7; 2.3 | -3.6 | -5.1; -2.1 |  |  |  |  |  |  |  |  |
| Maputo Cidade (MOZ, 2003-15) | -0.1 | -1.3; 1.0 | -1.9 | -3.8; 0.0 | 4.2 | 2.4; 6.0 | 6.7 | 6.0; 7.4 | -2.3 | -2.4; -2.2 | -0.1 | -0.1; -0.1 |
| Maseru (LSO, 2004-14) | -3.4 | -7.7; 0.8 | -6.0 | -10.9; -1.2 | 2.2 | -0.4; 4.7 | -1.2 | -16.9; 14.5 | 1.6 | -5.2; 8.4 | 1.4 | -1.8; 4.6 |
| N'Djaména (TCD, 2004-19) | -1.8 | -4.5; 0.9 | -1.0 | -3.2; 1.2 | 3.7 | 1.9; 5.5 | 0.7 | -0.1; 1.4 | -1.4 | -1.5; -1.3 | -2.3 | -2.3; -2.3 |
| Nairobi (KEN, 2003-14) | -3.5 | -7.6; 0.6 | -0.7 | -10.0; 8.7 | -1.8 | -10.1; 6.4 | 5.1 | 0.9; 9.4 | -4.2 | -9.4; 1.0 | -0.4 | -6.5; 5.7 |
| Niamey (NER, 2006-21) | -6.1 | -6.3; -5.8 | -1.0 | -2.0; 0.0 | 3.5 | 3.0; 4.0 | -0.9 | -9.7; 7.9 | -5.1 | -6.3; -3.8 | -4.3 | -9.1; 0.4 |
| Nouakchott (MRT, 2007-2019) | -1.8 | -4.2; 0.5 | -1.4 | -4.5; 1.7 |  |  |  |  |  |  |  |  |
| Ouagadougou (BFA, 2003-10) | 1.1 | -6.3; 8.6 | -1.5 | -11.6; 8.7 | 0.5 | -1.3; 2.3 | -12.0 | -12.7; -11.2 | -2.2 | -2.2; -2.1 | -4.9 | -4.9; -4.9 |
| SAB (GNB, 2006-18) | -5.9 | -6.9; -4.9 | -10.5 | -12.3; -8.8 |  |  |  |  |  |  |  |  |
| Ville D'Abidjan (CIV, 2006-16) | -9.8 | -15.1; -4.5 | -9.8 | -11.5; -8.1 |  |  |  |  |  |  |  |  |
| Windhoek (NAM, 2000-13) | -8.2 | -14.5; -1.9 | -7.8 | -13.3; -2.3 | -7.5 | -24.2; 9.2 | 7.5 | -0.2; 15.2 | -5.6 | -9.9; -1.3 | 8.9 | 0.4; 17.3 |
| **Median AARC and IQR** | **-2.2** | **-4.0; -1.5** | **-3.3** | **-5.9; -1.0** | **-1.3** | **-3.1; 1.0** | **-1.5** | **-4.8; 0.7** | **-3.3** | **-5.1; -1.9** | **-3.1** | **-4.9; -1.5** |
